# Auswirkungen der SARS-CoV-2-Pandemie auf die Chirurgie – Eine nationale Querschnittsstudie

**DOI:** 10.1007/s00104-020-01256-x

**Published:** 2020-08-10

**Authors:** Christian Stöß, Henryk Haffer, Marcella Steffani, Ilaria Pergolini, Daniel Hartmann, Ulrich Nitsche, Alexander Novotny, Helmut Friess, Michael W. Müller

**Affiliations:** 1 Klinik und Poliklinik für Chirurgie, Klinikum rechts der Isar, Fakultät für Medizin, Technische Universität München, Ismaninger Straße 22, 81675 München, Deutschland; 2 Centrum für Muskuloskeletale Chirurgie (CMSC), Charité – Universitätsmedizin Berlin, Berlin, Deutschland

**Keywords:** Deutsches Gesundheitssystem, Allgemein- und Viszeralchirurgie, Umstrukturierungen, Versorgungsqualität, Universitätsmedizin, German healthcare system, General and visceral surgery, Restructuring, Quality of care, University medicine

## Abstract

**Hintergrund und Ziele:**

Das deutsche Gesundheitssystem wurde mit Beginn der SARS-CoV-2-Pandemie auf die Behandlung von COVID-19-Patienten ausgerichtet. Dies ging mit der Aussetzung des operativen Elektivprogramms einher. Ziel der Studie war es, den Einfluss der SARS-CoV-2-Pandemie auf die Allgemein- und Viszeralchirurgie der Universitätskliniken in Deutschland zu untersuchen.

**Methoden:**

Die vorliegende Querschnittsstudie basiert auf einer anonymen Umfrage, die vom 03.04.2020 bis zum 17.04.2020 für die Teilnehmer des Konvents der Ordinarien für Allgemein- und Viszeralchirurgie in Deutschland online zugänglich war. 73 % (*n* = 29/40) der Befragten nahmen teil.

**Ergebnisse:**

Die Zusammenarbeit mit den Behörden und der Krankenhausleitung wurde überwiegend als angemessen empfunden. Allerdings stimmten nur 3 % (1/29) bzw. 7 % (2/29) der Aussage voll zu, dass sich die Gesundheitsbehörden auf Bundes- bzw. Landesebene unterstützend gegenüber der Allgemein- und Viszeralchirurgie zeigten. Die Klinikdirektoren erwarten einen niedrigeren Umsatz von durchschnittlich 28 ± 16 %. Es kam zu einer durchschnittlichen Verringerung der Betten- bzw. Operationssaalkapazität um 38 % bzw. 45 %. Darüber hinaus wurden 11 % des ärztlichen Personals der Allgemein- und Viszeralchirurgie in andere Abteilungen umverteilt.

**Diskussion:**

Die SARS-CoV-2-Pandemie hat deutliche Auswirkungen auf die universitäre Allgemein- und Viszeralchirurgie in Deutschland. Die Reduktion der Betten- sowie der Operationsaalkapazität kann zu erheblichen Verzögerungen dringlicher Operationseingriffe und finanziellen Belastungen im Jahr 2020 und den Folgejahren führen.

**Zusatzmaterial online:**

Zusätzliche Informationen sind in der Onlineversion dieses Artikels (10.1007/s00104-020-01256-x) enthalten.

## Abstract (en)

**Background and aims:**

From the beginning of the SARS-CoV‑2 pandemic the German healthcare system focused on the treatment of COVID-19 patients. This was accompanied by the suspension of all elective operations. The aim of this study was to investigate the impact of the SARS-CoV‑2 pandemic on general and visceral surgery in university hospitals in Germany.

**Methods:**

This cross-sectional study was based on an anonymous survey, which was accessible online from 3 April 2020 to 17 April 2020 for the surgical departments of university hospitals in Germany. In total 73% (*n* = 29/40) of the hospitals participated in the survey.

**Results:**

Cooperation with the authorities and the hospital administration was generally considered adequate; however, only 3% (1/29) and 7% (2/29) fully agreed with the statement that the health authorities at the federal and state level, respectively, were supportive of general and visceral surgery. The hospital directors expect an average loss of revenues of 28 ± 16%. There was an average reduction in beds or operating room capacity of 38% and 45%, respectively. In addition, 11% of the medical personnel in general and visceral surgery were reallocated to other departments.

**Conclusion:**

The SARS-CoV‑2 pandemic has a significant impact on academic general and visceral surgery in Germany. The reduction in beds and operating room capacity can lead to considerable delays in urgent surgical interventions and financial burdens in 2020 and subsequent years.

Die SARS-CoV-2-Pandemie breitet sich seit Dezember 2019 weltweit aus. Die Auswirkungen auf die Allgemein- und Viszeralchirurgie waren besonders deutlich: Um Krankenhauskapazitäten für COVID-19-Patienten bereitzustellen, mussten elektive Operationen ausgesetzt und verschoben werden. Nun dürfen die Bettenkapazitäten schrittweise wieder für elektive Eingriffe freigegeben und genutzt werden. In diesem Beitrag werden die Wahrnehmungen und Erfahrungswerte der Ordinarien für Allgemein- und Viszeralchirurgie in Deutschland in dieser frühen Pandemiephase vorgestellt.

## Hintergrund

Ende Dezember 2019 wurde in der Stadt Wuhan, China, erstmalig eine durch ein neuartiges Coronavirus (SARS-CoV-2) verursachte Lungenerkrankung beschrieben, die am 11.02.2020 von der World Health Organization (WHO) als „coronavirus disease 2019“ (COVID-19) bezeichnet wurde [1–3]. Am 11.03.2020 erklärte die WHO den COVID-19-Ausbruch zur Pandemie [4]. Im gleichen Monat wurden bundesweit weitreichende Eindämmungs- und Minderungsstrategien für die gesamte Bevölkerung beschlossen. Um Krankenhauskapazitäten, Schutzausrüstungen und Beatmungsgeräte für eine steigende Zahl von COVID-19-Patienten bereitzustellen, wurden die chirurgischen Fachdisziplinen angewiesen, alle elektiven Operationen zu verschieben und Personal bei Bedarf auf Intensiv- und COVID-19-Stationen umzuverteilen [5]. Die Deutsche Gesellschaft für Allgemein- und Viszeralchirurgie veröffentlichte am 22.03.2020 eine Stellungnahme mit einem Überblick dringlicher Operationseingriffe, die als noch durchführbar anzusehen waren [6]. Auch andere operative Fachbereiche waren von den Umstrukturierungen betroffen [7–9]. Ende April nach Abschluss der vorliegenden Befragung beschloss die deutsche Bundesregierung, dass die Krankenhauskapazitäten schrittweise wieder für elektive Operationen genutzt werden dürfen. Die langfristigen Auswirkungen der Aussetzung des Elektivprogramms auf die Allgemein- und Viszeralchirurgie der Universitätskliniken in Deutschland sind zum aktuellen Zeitpunkt noch nicht absehbar.

Die vorliegende Querschnittsstudie hat zum Ziel, den Einfluss der globalen SARS-CoV-2-Pandemie auf die Allgemein- und Viszeralchirurgie der Universitätskliniken in Deutschland zu evaluieren. In der durchgeführten Umfrage wurden Daten zu Erfahrungen bezüglich personeller Umstrukturierungen und finanziellen Belastungen sowie zur Einschätzung der zukünftigen Entwicklung erhoben.

## Methoden

### Studiendesign

Für die vorliegende Querschnittsstudie wurden vom 03. bis 17.04.2020 alle Teilnehmer des Konvents der Ordinarien elektronisch zu einer anonymen Onlineumfrage eingeladen. Dies entspricht 40 Lehrstuhlinhabern für Allgemein- und Viszeralchirurgie der deutschen Universitätsklinika. Die Teilnahme an der Umfrage war freiwillig, personenbezogene Daten wurden nicht erhoben.

### Umfrage

Für die Onlineumfrage wurde ein kommerzieller Anbieter genutzt (Google Formulare, https://docs.google.com/forms; Google Inc. Mountain View, CA, USA). Die Umfrage umfasste 67 Einzelfragen und Aussagen in 8 Kategorien (allgemeine Informationen, Politik, Gesundheitsbehörden, Krankenhausleitung, Kommunikation mit anderen Fachbereichen, Folgen der Umstrukturierungen, Auswirkungen auf die Fallzahlen, Ausblick auf die Zeit nach der SARS-CoV-2-Pandemie). Es wurde eine Kombination aus bipolaren, nummerierten Likert-Skalen (1 entspricht „stimme voll zu“, 2 entspricht „stimme zu“, 3 entspricht „neutral“, 4 entspricht „stimme nicht zu“ und 5 entspricht „stimme überhaupt nicht zu“), geschlossenen (ja/nein/unbekannt) und offenen Fragen verwendet.

Im 1. Abschnitt wurden bei den Befragten allgemeine Charakteristiken (9 Fragen) erhoben. Im 2. (Politik, 5 Fragen) und 3. Abschnitt (Gesundheitsbehörden, 4 Fragen) wurden die Befragten gebeten, die Kommunikation und die von der Bundes- und Landesregierung sowie den Gesundheitsbehörden ergriffenen Maßnahmen zur Eindämmung der SARS-CoV-2-Pandemie zu bewerten. In der 4. Kategorie (Krankenhausleitung/Klinikvorstand, 8 Fragen) wurde die Zusammenarbeit mit der Krankenhausverwaltung erfragt. Die 5. Kategorie (Zusammenarbeit mit anderen Fachabteilungen während der SARS-CoV-2-Pandemie, 5 Fragen) untersuchte die Zusammenarbeit mit den anderen Fachabteilungen und insbesondere die Fortführung des interdisziplinären Tumorboards. In der 6. und 7. Kategorie (Umstrukturierung/Pandemiefolgen, 28 Fragen) wurden jeweils die bisherigen Auswirkungen der SARS-CoV-2-Pandemie bewertet. Es wurde ein Schwerpunkt auf die 6. und 7. Kategorie gelegt, da die Aussetzung aller elektiven Operationen und die Neuzuweisung von Personal für die Betreuung der COVID-19-Patienten einen erheblichen strukturellen Einfluss auf die Abteilungen haben. Zum Abschluss wurden die Befragten in der 8. Kategorie (Ausblick für die Zeit nach der SARS-CoV-2-Pandemie, 8 Fragen) gebeten, mögliche Veränderungen durch die Pandemie in der Zukunft zu bewerten. Die vollständige Umfrage findet sich im Supplement dieses Beitrages (s. Zusatzmaterial online).

### Statistische Analyse

Die deskriptive Datenanalyse wurde mit Microsoft Excel 2019 (Microsoft, Redmond, WA, USA) durchgeführt. Die Angaben erfolgen als absolute sowie relative Zahlen und als Mittelwert (± Standardabweichung).

## Ergebnisse

### Allgemeine Charakteristiken

Insgesamt nahmen 73 % der Befragten an der Umfrage teil. Die Teilnehmer waren zu 93 % männlich. Acht (28 %) der 29 Befragten waren unter 50 Jahre alt, 21 (72 %) waren älter als 50 Jahre. Hinsichtlich der Krankenhausgröße (Anzahl an Betten) gaben 20 der 29 Befragten (69 %) eine Größe von über 1000 Betten an. Sieben (24 %) gaben an, unter 50 Intensivbetten im Krankenhaus zur Verfügung zu haben. Zehn (34 %) bzw. 12 (41 %) gaben eine Gesamtzahl verfügbarer Intensivbetten von 51 bis 100 bzw. über 100 an. Zwei der 29 Teilnehmer (7 %) gaben weniger als 2000 allgemein- und viszeralchirurgische Operationen pro Jahr an, wohingegen 25 (86 %) mit 2000 bis 4000 Eingriffen antworteten und 2 (7 %) von über 4000 Eingriffen pro Jahr berichteten (Tab. 1).

**Table 1:** 

Charakteristika		Anzahl der Teilnehmer (%)
*Gesamt*		29 (100)
*Alter (Jahre)*	≤50	8 (28)
	>50	21 (72)
*Geschlecht*	Männlich	27 (93)
	Weiblich	2 (7)
*Krankenhausgröße (Anzahl an Betten)*	≤1000	9 (31)
	>1000	20 (69)
*Intensivkapazität (Anzahl an Intensivbetten)*	<50	7 (24)
	51–100	10 (34)
	>100	12 (41)
*Anzahl an jährlich durchgeführten Operationen*	<2000	2 (7)
	2000–4000	25 (86)
	>4000	2 (72)

### Wahrnehmung der Politik und Kooperation mit den Behörden

**Figure 1:**
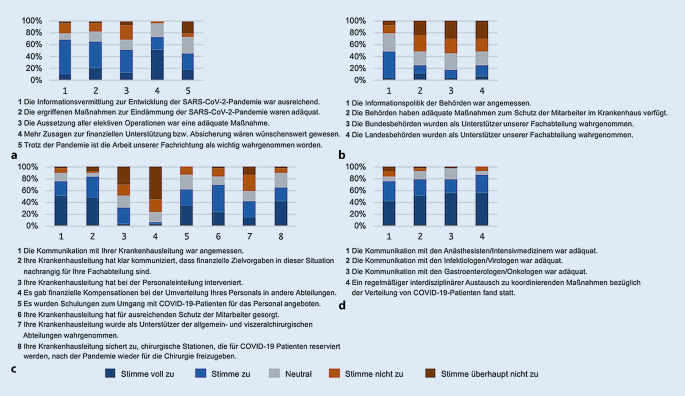

Der Aussage, dass die Informationsvermittlung der Politik zur SARS-CoV-2-Pandemie ausreichend war, stimmten 10 % und 59 % der Klinikdirektoren voll zu bzw. zu (Abb. 1). Die Informationspolitik der Gesundheitsbehörden empfanden 48 % der Befragten als angemessen (3 % stimmten voll zu, 45 % stimmten zu). Auch der Aussage, dass die Maßnahmen zur Eindämmung der Pandemie adäquat waren, stimmte die Mehrheit zu: 21 % stimmten voll zu und 45 % stimmten zu. Der Aussetzung der elektiven Operationen als adäquate Maßnahme stimmten 14 % voll zu und 38 % stimmten zu, hingegen stimmten 24 % nicht zu und 7 % stimmten überhaupt nicht zu. Mehrheitlich zeigte sich, dass sich die Klinikdirektoren mehr finanzielle Unterstützung gewünscht hätten: Insgesamt 52 % stimmten voll und 21 % stimmten der Aussage zu.

### Interne Kommunikation mit der Krankenhausleitung

Insgesamt 48 % der Teilnehmer stimmten voll zu und 34 % stimmten zu, dass die Krankenhausleitung zuvor vereinbarte finanzielle Zielvorgaben aktuell als nachrangig ansieht (Abb. 1). Insgesamt 10 % stimmten dieser Aussage nicht zu. Der Aussage, dass es finanzielle Kompensationen bei der Umverteilung des Personals gab, stimmten 3 % voll zu und 3 % stimmten zu, jedoch stimmten insgesamt 76 % der Befragten nicht zu. Bei 30 % der Befragten intervenierte die Krankenhausleitung bei der Personaleinteilung, bei insgesamt 76 % war dies nicht der Fall. 41 % stimmten voll und 24 % stimmten zu, dass ihre Krankenhausleitung zugesichert hat, dass die vorgehaltenen Betten nach der Pandemie wieder für die Chirurgie freigegeben werden.

### Zusammenarbeit mit anderen Fachabteilungen

Zwischen 75 und 86 % der Befragten stimmten den jeweiligen Aussagen zu, einen interdisziplinären Austausch mit der Anästhesie, Intensivmedizin, Infektiologie, Virologie, Onkologie und der Gastroenterologie zu führen (Abb. 1). Das interdisziplinäre Tumorboard wurde in 10 % der Fälle wie vor der Pandemie durchgeführt, 55 % der Befragten gaben an, das Tumorboard mit weniger Personal und 34 % per Videokonferenz durchzuführen. In keinem Klinikum wurde das Tumorboard pausiert (Supplement Abb. 1, s. Zusatzmaterial online).

### Auswirkungen der Pandemie auf die Viszeralchirurgie

In der Umfrage zeigte sich, dass aufgrund der SARS-CoV-2-Pandemie die Bettenkapazität der universitären Allgemein- und Viszeralchirurgie vorübergehend im Mittel um 38 ± 19 % reduziert wurde (Tab. 2). Die Bettenauslastung wurde dabei mit 62 ± 23 % angegeben. Die Operationssaalkapazität wurde um durchschnittlich 45 ± 16 % reduziert. Die Auslastung der reduzierten Operationsaalkapazität lag im Mittel bei 69 ± 28 %. Auch die Kapazität der Sprechstunden war mit einer Reduktion von durchschnittlich 59 ± 25 % eingeschränkt. Laut Studienteilnehmern waren auch andere operative Fachabteilungen von einer Reduktion der Betten- sowie Operationsaalkapazität betroffen. Der erwartete Verlust für den Umsatz 2020 wurde im Mittel bei 28 ± 16 % angegeben. 21 % der Studienteilnehmer denken, dass dies finanzielle Konsequenzen für die eigene Abteilung hat und 10 % stimmten zu, dass sich dies auf die personelle Situation auswirken wird (Tab. 3).

**Table 2:** 

Fragen	Antwort (in %) Mittelwert ± Standardabweichung
Schätzen Sie die aktuelle Reduktion der Bettenkapazität Ihrer Abteilung als Folge der Pandemie.	38 (±19)
Schätzen Sie die aktuelle Bettenauslastung Ihrer Abteilung als Folge der Pandemie.	62 (±23)
Schätzen Sie die aktuelle Reduktion der Operationssaalkapazität Ihrer Abteilung als Folge der Pandemie.	45 (±16)
Schätzen Sie die aktuelle Operationssaalauslastung Ihrer Abteilung als Folge der Pandemie.	69 (±28)
Schätzen Sie die aktuelle Reduktion der Sprechstundenkapazität Ihrer Abteilung als Folge der Pandemie.	59 (±25)
Schätzen Sie die aktuelle Auslastung der Sprechstunden Ihrer Abteilung als Folge der Pandemie.	46 (±26)
Schätzen Sie den Anteil der ärztlichen Mitarbeiter, die von Ihrer Abteilung zu anderen Abteilungen umverteilt werden sollen.	11 (±12)
Schätzen Sie den Anteil der ärztlichen Mitarbeiter Ihrer Abteilung in „Kurzarbeit“ oder „Schichtarbeit“.	11 (±16)
Schätzen sie den Anteil der ärztlichen Mitarbeiter Ihrer Abteilung, die sich mit SARS-CoV‑2 infiziert haben.	1 (±2)
Schätzen Sie den Verlust Ihrer Zielvorgaben für den Umsatz 2020.	28 (±16)
Schätzen Sie den Verlust Ihrer Zielvorgaben für die Case-Mix-Punkte.	26 (±12)
Schätzen Sie den Verlust Ihrer Zielvorgaben für den Case-Mix-Index.	20 (±17)

**Table 3:** 

Fragen	Antworten (*n* = 29)		
	Ja *n* (%)	Nein *n* (%)	Unbekannt *n* (%)
Wurde von Ihrer Klinikleitung Überstundenabbau angeordnet?	13 (45)	16 (55)	0
Wurde von Ihrer Klinikleitung Urlaub angeordnet?	1 (3)	27 (93)	1 (3)
Konnte ausreichend angemessene Schutzausrüstung für Ihr Personal zur Verfügung gestellt werden?	21 (72)	7 (24)	1 (3)
Mussten Notfalloperationen aufgrund krankheitsbedingten Ausfalls verschoben bzw. verlegt werden?	2 (7)	27 (93)	0
Würden Sie zum aktuellen Zeitpunkt schätzen, dass ein Nichterreichen der Zielvorgaben finanzielle Konsequenzen für Ihre Abteilung hat?	6 (21)	15 (52)	7 (24)
Würden Sie zum aktuellen Zeitpunkt schätzen, dass ein Nichterreichen der Zielvorgaben personelle Konsequenzen für Ihre Abteilung hat?	3 (10)	16 (55)	10 (34)
Wurde die Bettenkapazität auch in anderen operativen Fachabteilungen reduziert?	27 (93)	0	2 (7)
Wurde die Operationssaalkapazität auch in anderen operativen Fachabteilungen reduziert?	29 (100)	0	0

Den Schutz der Mitarbeiter betreffend äußerten 24 %, dass nicht ausreichend genug Schutzkleidung zur Verfügung stand. Bei 7 % der Befragten mussten Notfalloperationen verschoben oder verlegt werden. 93 % der Studienteilnehmer waren überzeugt, dass Patienten aus Sorge vor einer COVID-19-Infektion elektive Operationen absagten (Tab. 4). 26 % stimmten zu, dass die Anzahl der Notfalloperationen gesunken ist, bei 56 % war diese gleich und bei 19 % stieg die Anzahl an Notoperationen an. Die Zahl der chirurgischen Notfälle in der Notaufnahme hatte bei 36 % der Befragten abgenommen, bei 11 % zugenommen und 54 % gaben an, eine gleiche Anzahl von Notfällen zu behandeln. Es zeigte sich eine deutliche Auswirkung der Pandemie auf die Behandlung onkologischer Patienten: 65 % der Befragten gaben an, weniger onkologische Patienten in der Sprechstunde zu behandeln, lediglich 27 % gaben eine gleichbleibende Anzahl an.

**Table 4:** 

Frage	*n* (Gesamt)	Antwort	*n* (%)	Änderung (in %) Mittelwert ± Standardabweichung
Die Anzahl an Notfalloperationen ist …	27	*Gestiegen*	5 (19)	27 ± 18
		*Gleich geblieben*	15 (56)	–
		*Gesunken*	7 (26)	33 ± 11
Die Anzahl an chirurgischen Notfällen in der Notaufnahme ist …	28	*Gestiegen*	3 (11)	23 ± 6
		*Gleich geblieben*	15 (54)	–
		*Gesunken*	10 (36)	34 ± 11
Die Anzahl an onkologischen Patienten in der Sprechstunde ist …	26	*Gestiegen*	2 (8)	20 ± 0
		*Gleich geblieben*	7 (27)	–
		*Gesunken*	17 (65)	42 ± 22
Haben Sie den Eindruck, dass Patienten aus Sorge vor einer SARS-CoV-2-Infektion elektive Operationen absagen?	29	*Ja*	27 (93)	–
		*Nein*	0	–
		*Unbekannt*	2 (7)	–

### Ausblick auf die Zeit nach der SARS-CoV2-Pandemie

**Figure 2:**
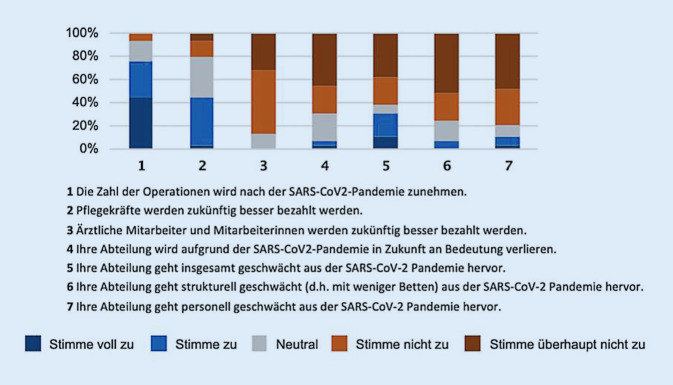

Die Studienteilnehmer wurden zu den Auswirkungen der Pandemie auf die Bezahlung des Pflege- und Ärztepersonals befragt. Etwa 44 % der Ordinarien gehen davon aus, dass vor allem Pflegekräfte künftig besser bezahlt werden. Hingegen stimmte keiner der Aussage zu, dass es zukünftig eine bessere Bezahlung für das ärztliche Personal geben werde. 10 % stimmten voll und 21 % stimmten zu, dass die Allgemein- und Viszeralchirurgie geschwächt aus der SARS-CoV-2-Pandemie hervorgehen wird, wohingegen insgesamt 62 % der Aussage nicht zustimmten (Abb. 2). 76–79 % der Studienteilnehmer gingen davon aus, dass es nicht zu Veränderungen des Personals und der Bettenanzahl kommen wird und lediglich 7–10 % der Befragten gingen davon aus, in Bezug auf diese Parameter nach der Pandemie geschwächt zu sein.

## Diskussion

Die vorliegende Querschnittsstudie liefert erste Daten zur Auswirkungen der SARS-CoV-2-Pandemie auf die Allgemein- und Viszeralchirurgie der Universitätskliniken während der Aussetzung aller Elektivoperationen. Die Teilnahmerate von 73 % weist auf die große Bedeutung der Thematik für die Ordinarien der Allgemein- und Viszeralchirurgie hin.

Die Ergebnisse zeigen, dass die Maßnahmen zur Eindämmung der SARS-CoV-2-Pandemie überwiegend positiv seitens der Ordinarien aufgenommen wurden. Die Zusammenarbeit der Allgemein- und Viszeralchirurgie mit der Krankenhausverwaltung und anderen Fachdisziplinen erfolgte überwiegend erfolgreich. Hervorzuheben sind auch die Ergebnisse zur Nachrangigkeit der finanziellen Zielvorgaben. Die Arbeit der Gesundheitsbehörden im Hinblick auf den Schutz der Mitarbeiter in den Krankenhäusern wurde überwiegend als ausreichend empfunden. So herrscht aufgrund der SARS-CoV-2-Pandemie weltweit in immer mehr Gesundheitseinrichtungen ein Mangel an wichtiger Ausrüstung wie Desinfektionsmittel und persönlicher Schutzausrüstung [10]. 24 % der Befragten gaben an, dass zunächst nicht ausreichend genug Schutzausrüstung zur Verfügung stand. Daraus folgt die Erkenntnis und Forderung, Reserven für künftige Pandemien aufzubauen, um sie zügiger bereitstellen zu können.

Die chirurgischen Kliniken wurden am 16. März 2020 aufgefordert, alle medizinisch nicht zwingend notwendigen planbaren Aufnahmen und Operationen zu verschieben [11]. Ab Mai durfte das allgemeine Operationsprogramm schrittweise wieder aufgenommen werden [12]. Für die chirurgischen Fachdisziplinen bedeutet diese Pandemiephase eine gravierende finanzielle Einbuße. Zur Finanzierung dieser Einbuße wurde das „COVID-19-Krankenhausentlastungsgesetz“ verabschiedet [13]. Inwieweit jedoch damit die finanziellen Belastungen konkret ausgeglichen werden können, bleibt abzuwarten. Insgesamt kam es zur durchschnittlichen Reduktion der Betten- bzw. Operationskapazität um 38 ± 19 % bzw. 45 ± 16 %. Die Reduktion der chirurgischen Kapazitäten ließ die Befragten einen Verlust der Einnahmen im Jahr 2020 von 28 ± 16 % schätzen. Auch nach Rückbau der Maßnahmen könnten durch strukturelle Gegebenheiten bei Personal und Betten- bzw. Operationskapazität diese Verluste nicht aufgeholt werden. Dies gilt nicht nur für die Allgemein- und Viszeralchirurgie. Die Befragten nahmen dies auch für andere chirurgische Fachbereiche wahr. Aufgrund der erheblichen Einschränkung der operativen Kapazitäten und der Umverteilung von Personal wurden in den meisten Einrichtungen nur noch dringliche Operationen und Notfalloperationen durchgeführt, wobei die Durchführung von Notfalloperationen an den Universitätskliniken in 93 % der Fälle uneingeschränkt funktionierte. Die Klinikdirektoren der universitären Allgemein- und Viszeralchirurgie gaben einen Rückgang der onkologischen Patienten in den Sprechstunden an. Die Mehrheit der Lehrstuhlinhaber berichtete zudem von einer zunehmenden Verunsicherung der Patienten mit der Folge von Absagen und Verschiebungen von Operationen. Dies kann zur Verschlechterung der onkologischen Behandlungsqualität durch zeitliche Verzögerung führen. Darüber hinaus sollte bedacht werden, dass nach Wiederaufnahme des Elektivprogramms die verschobenen Operationen zusätzlich durchgeführt werden müssen. Es bedarf somit bereits jetzt einer suffizienten Planung, wie verschobene Operationen zusätzlich zum Elektivprogramm ohne weitere Zeitverzögerung stattfinden können. Außerdem ist abgezogenes Personal aus den vorgehaltenen COVID-19-Bereichen zurückzuführen, um die erforderliche Bettenkapazität zu gewährleisten.

Die Ordinarien gehen davon aus, dass Pflegekräfte zukünftig besser bezahlt werden. Eher gleichbleibend zeigt sich die Einschätzung für das ärztliche Personal. Aus Sicht der Ärzteschaft ist dies ein positives Signal hin zur Stärkung der Pflege. Bereits vor der SARS-CoV-2-Pandemie war zum einen die geringe Vergütung dieser systemrelevanten Berufsgruppe und zum anderen der prognostizierte Fachkräftemangel im Pflegesektor diskutiert worden [14, 15]. Die jetzige Situation könnte als Wendepunkt dienen, um den Pflegebereich nachhaltig zu stärken. Es bleibt zu hoffen, dass nach Rückbau der COVID-19-Kapazitäten noch genügend Pflegekräfte zur Verfügung stehen, da in der Pandemiehochphase die Neurekrutierung nicht im Vordergrund stand und es weiterhin schwierig bleibt, in Ballungsgebieten ausreichend Pflegepersonal zu finden.

Die vorliegende Querschnittsstudie geht mit Limitationen einher. Die erhobenen Daten basieren auf subjektiven Einschätzungen der Ordinarien. Der Befragungszeitraum erfolgte während des vorerst ersten Höhepunkts der SARS-CoV-2-Pandemie, sodass die Ergebnisse als vorläufig zu betrachten sind und die weitere Entwicklung bzw. die abschließenden Auswirkungen in geplanten Folgeumfragen evaluiert werden sollten.

Unseres Wissens ist dies bislang die erste Umfrage, die die Einflüsse der SARS-CoV-2-Pandemie auf die Allgemein- und Viszeralchirurgie der deutschen Universitätsklinika umfassend darstellt und somit einen Überblick über die Herausforderungen in der universitären, chirurgischen Versorgungsrealität gibt. Während des Befragungszeitraums befand sich Deutschland noch in einem frühen Stadium der SARS-CoV-2-Pandemie, sodass zukünftige Auswirkungen derzeit noch schwer absehbar sind. Offen bleibt, ob die Reduktion der Operationskapazität und das Aussetzen der elektiven Operationen zu einer Minderung der chirurgischen Versorgungsqualität in Deutschland führen und ob es zu Nachteilen in der Versorgung und den Ergebnissen bei onkologischen Patienten kommen wird. Folgeerhebungen müssen hierzu zukünftig weitere Evidenz liefern. Betrachtet man die Ergebnisse zur Bereitstellung der Schutzausrüstung für das Personal, zur Bezahlung der Pflegekräfte und zu den finanziellen Belastungen für die Kliniken, muss man von der Politik Lösungsvorschläge erwarten. Dabei sollte auch eine mögliche nächste SARS-CoV-2-Pandemiewelle berücksichtigt werden, die nach Lockerung der Eindämmungsmaßnahmen möglich erscheint. Der aktuelle Rückbau in den Krankenhäusern ohne vorhandenen Impfstoff muss mit Plänen für den Fall steigender Infektionszahlen einhergehen. Dabei muss das Ausmaß der Vorhaltungen für Universitätsklinika und nichtuniversitäre Krankenhäuser präzise definiert werden, um Planungssicherheiten zu bieten. Zudem steht zur Diskussion, ob eine Konzentrierung von COVID-19-Patienten zukünftig auf ausgewählte Krankenhäuser erfolgen sollte, um die Versorgung aller anderen Patienten gewährleisten zu können.

## Fazit

- Bei einem Viertel der Befragten stand zu Beginn der Pandemie zu wenig Schutzausrüstung zur Verfügung. Zukünftig sollten Reserven für weitere Pandemiewellen bereit stehen.
- Die Betten- bzw. Operationskapazität wurde um 38 % bzw. 45 % reduziert. Die verschobenen Operationen werden zusätzlich zum wiederaufgenommenen Elektivprogramm anfallen und bewältigt werden müssen.
- Die Ordinarien schätzen den Verlust der Einnahmen im Jahr 2020 auf ca. 28 %. Eine Kompensation dieses Verlusts aus eigener Kraft scheint nur schwer möglich.
- Die chirurgische Versorgungsqualität ist aktuell kaum eingeschränkt, nur 7 % der Notfalloperationen an den befragten Universitätskliniken konnten nicht durchgeführt werden.
- Die chirurgischen Kliniken benötigen weitere Planungssicherheiten, um während der anhaltenden Maßnahmen zur Eindämmung der SARS-CoV-2-Pandemie die chirurgische Versorgung der Patienten zu gewährleisten.

## Caption Electronic Supplementary Material






## References

[1] Zhu N (2020). A novel Coronavirus from patients with pneumonia in China, 2019. N Engl J Med.

[2] Lu R (2020). Genomic characterisation and epidemiology of 2019 novel coronavirus: implications for virus origins and receptor binding. Lancet.

[3] Rothan HA, Byrareddy SN (2020). The epidemiology and pathogenesis of coronavirus disease (COVID-19) outbreak. J Autoimmun.

[4] Cucinotta D, Vanelli M (2020). WHO declares COVID-19 a pandemic. Acta Biomed.

[5] Bundesministerium für Gesundheit. Coronavirus SARS-CoV-2: Chronik der bisherigen Maßnahmen. https://www.bundesgesundheitsministerium.de/coronavirus/chronik-coronavirus.html. Zugegriffen: 6. Mai 2020

[6] DGAV Rundschreiben zu OP-Indikationen. https://www.dgav.de/dgav/aktuelles/aktuelles-aus-der-dgav/article/dgav-rundschreiben-zu-op-indikationen.html. Zugegriffen: 3. Mai 2020

[7] Liebensteiner MC et al (2020) Massive cutback in orthopaedic healthcare services due to the COVID-19 pandemic. Knee Surg Sports Traumatol Arthrosc. 10.1007/s00167-020-06032-2 10.1007/s00167-020-06032-2 PMC7192059 32356047

[8] Ducournau F (2020). COVID-19: initial experience of an international group of hand surgeons. Hand Surg Rehabil.

[9] Fontanella MM et al (2020) Neurosurgical activity during COVID-19 pandemic: an expert opinion from China, South Korea, Italy, United Stated of America, Colombia and United Kingdom. J Neurosurg Sci. 10.23736/S0390-5616.20.04994-2 10.23736/S0390-5616.20.04994-2 32347685

[10] Feng S (2020). Rational use of face masks in the COVID-19 pandemic. Lancet Respir Med.

[11] Die Bundesregierung. Besprechung der Bundeskanzlerin mit den Regierungschefinnen und Regierungschefs der Länder am 12. März 2020. https://www.bundesregierung.de/breg-de/themen/coronavirus/beschluss-zu-corona-1730292. Zugegriffen: 16. Mai 2020

[12] Bundesministerium für Gesundheit Ein neuer Alltag auch für den Klinikbetrieb in Deutschland. https://www.bundesgesundheitsministerium.de/fileadmin/Dateien/3_Downloads/C/Coronavirus/Faktenpapier_Neuer_Klinikalltag.pdf. Zugegriffen: 16. Mai 2020

[13] Bundesministerium für Gesundheit. Ein neuer Alltag auch für den Klinikbetrieb in Deutschland. https://www.bundesgesundheitsministerium.de/presse/pressemitteilungen/2020/1-quartal/gesetzespakete-corona-epidemie.html. Zugegriffen: 6. Mai 2020

[14] Afentakis A, Maier T (2010). Projektionen des Personalbedarfs und -angebots in Pflegeberufen bis 2025. Wirtsch Stat.

[15] Afentakis A, Maier T (2013). Can nursing staff from abroad meet the growing demand for care? Analysis of labor migration in nursing professions in 2010. Bundesgesundheitsblatt Gesundheitsforschung Gesundheitsschutz.

