# Supplementary material for: Effects of the SARS-CoV-2 pandemic on surgery – a national cross-sectional study
Source: Chirurg. 2020 Aug 10;91(9):762–8. [Article in German] doi: 10.1007/s00104-020-01256-x (PMC7416587; doi:10.1007/s00104-020-01256-x)
Supplement: Supplementary file 1 [file 104_2020_1256_MOESM1_ESM.docx]

**Fragebogen**

**Allgemeine Informationen**

**1. Ihr Alter**

> 50 Jahre ☐

30-50 Jahre ☐

< 30 Jahre ☐

Keine Angabe ☐

**2. Geschlecht**

Männlich ☐

Weiblich ☐

Divers ☐

Keine Angabe ☐

**4. Berufsgruppe (obligat)**

Chefarzt/Chefärztin ☐

Leitender Oberarzt/leitende Oberärztin ☐

Oberarzt/Oberärztin ☐

Facharzt/Fachärztin ☐

**5. Versorgungsstufe des Krankenhauses**

Universitätsklinik ☐

Nicht universitäres Krankenhaus der Maximalversorgung ☐

Krankenhaus der Schwerpunkt- bzw. Zentralversorgung ☐

Krankenhaus der Grund- und Regelversorgung ☐

**6. Krankenhausart**

Privater Träger ☐

Öffentlich-rechtlicher Träger ☐

Freigemeinnütziger Träger ☐

**7. Krankenhausgröße (Anzahl der Betten)**

< 100 ☐

101-200 ☐

201-500 ☐

501-1000 ☐

> 1000 ☐

Keine Angabe ☐

**8. Intensivkapazität (Anzahl der Intensivbetten in Ihrem Krankenhaus)**

< 10 ☐

11-20 ☐

21-50 ☐

51-100 ☐

> 100 ☐

Keine Angabe ☐

**9. Bitten geben Sie die Anzahl der jährlich durchgeführten Operationen Ihrer**

**chirurgischen Abteilung an.**

**Politik (Bundes- und Landesebene)**

**10. Die Informationsvermittlung zur Entwicklung der SARS-CoV-2-Pandemie der**

**Politik war ausreichend.**

(1) Stimme voll zu (2) (3) (4) (5) Stimme überhaupt nicht zu

**11. Die ergriffenen Maßnahmen zur Eindämmung der SARS-CoV-2-Pandemie der**

**Politik waren adäquat.**

(1) Stimme voll zu (2) (3) (4) (5) Stimme überhaupt nicht zu

**12. Die Aussetzung aller elektiven Operationen war eine adäquate Maßnahme.**

(1) Stimme voll zu (2) (3) (4) (5) Stimme überhaupt nicht zu

**13. Mehr Zusagen zur finanziellen Unterstützung bzw. Absicherung wären**

**wünschenswert gewesen.**

(1) Stimme voll zu (2) (3) (4) (5) Stimme überhaupt nicht zu

**14. Trotz der Pandemie ist die Arbeit unserer Fachrichtung als wichtig**

**wahrgenommen worden.**

(1) Stimme voll zu (2) (3) (4) (5) Stimme überhaupt nicht zu

**Behörden (Robert Koch-Institut, Gesundheitsamt, Paul-Ehrlich-Institut)**

**15. Die Informationspolitik der Behörden war angemessen.**

(1) Stimme voll zu (2) (3) (4) (5) Stimme überhaupt nicht zu

**16. Die Behörden haben adäquate Maßnahmen zum Schutz der Mitarbeiter im**

**Krankenhaus verfügt (z. B.: Schutz in der Notaufnahme/OP/ Station).**

(1) Stimme voll zu (2) (3) (4) (5) Stimme überhaupt nicht zu

**17. Die Bundesbehörden wurden als Unterstützer unserer Fachabteilung**

**wahrgenommen.**

(1) Stimme voll zu (2) (3) (4) (5) Stimme überhaupt nicht zu

**18. Die Landesbehörden wurden als Unterstützer unserer Fachabteilung**

**wahrgenommen.**

(1) Stimme voll zu (2) (3) (4) (5) Stimme überhaupt nicht zu

**Krankenhausleitung / Klinikvorstand**

**19. Die Kommunikation mit Ihrer Krankenhausleitung war angemessen.**

(1) Stimme voll zu (2) (3) (4) (5) Stimme überhaupt nicht zu

**20. Ihre Krankenhausleitung hat klar kommuniziert, dass finanzielle Zielvorgaben in**

**dieser Situation nachrangig für Ihre Fachabteilung sind.**

(1) Stimme voll zu (2) (3) (4) (5) Stimme überhaupt nicht zu

**21. Ihre Krankenhausleitung hat bei der Personaleinteilung interveniert.**

(1) Stimme voll zu (2) (3) (4) (5) Stimme überhaupt nicht zu

**22. Es gab finanzielle Kompensationen bei der Umverteilung Ihres Personals in**

**andere Abteilungen (z. B.: Anästhesie/Intensivmedizin).**

(1) Stimme voll zu (2) (3) (4) (5) Stimme überhaupt nicht zu

**23. Es wurden rechtzeitig Schulungen zum Umgang mit COVID-19-Patienten für das**

**Personal angeboten.**

(1) Stimme voll zu (2) (3) (4) (5) Stimme überhaupt nicht zu

**24. Ihre Krankenhausleitung hat für ausreichenden Schutz der Mitarbeiter gesorgt**

**(z. B.: Erstellung von SOPs, Bereitstellung von Schutzausrüstung).**

(1) Stimme voll zu (2) (3) (4) (5) Stimme überhaupt nicht zu

**25. Ihre Krankenhausleitung wurde als Unterstützer der allgemein- und**

**viszeralchirurgischen Abteilungen wahrgenommen.**

(1) Stimme voll zu (2) (3) (4) (5) Stimme überhaupt nicht zu

**26. Ihre Krankenhausleitung sichert zu, chirurgische Stationen, die für COVID-19**

**Patienten reserviert werden, nach der Pandemie wieder für die Chirurgie**

**freizugeben.**

(1) Stimme voll zu (2) (3) (4) (5) Stimme überhaupt nicht zu

**Zusammenarbeit mit anderen Fachabteilungen im Rahmen der SARS-CoV-2-**

**Pandemie**

**27. Die Kommunikation mit den Anästhesisten / Intensivmedizinern war adäquat.**

(1) Stimme voll zu (2) (3) (4) (5) Stimme überhaupt nicht zu

**28. Die Kommunikation mit den Infektiologen / Virologen war adäquat.**

(1) Stimme voll zu (2) (3) (4) (5) Stimme überhaupt nicht zu

**29. Die Kommunikation mit den Gastroenterologen / Onkologen war adäquat.**

(1) Stimme voll zu (2) (3) (4) (5) Stimme überhaupt nicht zu

**30. Ein regelmäßiger interdisziplinärer Austausch zu koordinierenden Maßnahmen**

**bezüglich der Verteilung von COVID-19-Patienten fand statt.**

(1) Stimme voll zu (2) (3) (4) (5) Stimme überhaupt nicht zu

**31. Das interdisziplinäre Tumorboard findet weiterhin statt.**

Ja, wie bisher. ☐

Ja, aber mit weniger Personal. ☐

Ja, aber nur per Video-/Telefonkonferenz. ☐

Nein, das Tumorboard ist aktuell pausiert/reduziert. ☐

Keine Angabe ☐

**Umstrukturierung / Pandemiefolgen**

**32. Schätzen Sie die aktuelle Reduktion der Bettenkapazität (in %) Ihrer Abteilung als**

**Folge der Pandemie.** *(Sollte sich die Bettenkapazität Ihrer Klinik erhöht haben oder gleich geblieben sein, so tragen Sie hier bitte eine "0" ein.)*

**33. Schätzen Sie die aktuelle Bettenauslastung (in %) Ihrer Abteilung als Folge der**

**Pandemie.**

**34. Schätzen Sie die aktuelle Reduktion der OP-Saalkapazität (in %) Ihrer Abteilung**

**als Folge der Pandemie.** *(Sollte sich die OP-Saalkapazität Ihrer Klinik erhöht haben oder gleich geblieben sein, so tragen Sie hier bitte eine "0" ein.)*

**35. Schätzen Sie die aktuelle OP-Saalauslastung (in %) Ihrer Abteilung als Folge der**

**Pandemie.**

**36. Schätzen Sie die aktuelle Reduktion der Sprechstundenkapazität (in %) Ihrer**

**Abteilung als Folge der Pandemie.** *(Sollte sich die Sprechstundenkapazität Ihrer Klinik erhöht haben oder gleich geblieben sein, so tragen Sie hier bitte eine "0" ein.)*

**37. Schätzen Sie die aktuelle Auslastung der Sprechstunden (in %) Ihrer Abteilung**

**als Folge der Pandemie.**

**38. Wurde die Bettenkapazität auch in anderen operativen Fachabteilungen**

**reduziert?**

Ja ☐

Nein ☐

Nicht bekannt ☐

**39. Wurde die OP-Saalkapazität auch in anderen operativen Fachabteilungen**

**reduziert?**

Ja ☐

Nein ☐

Nicht bekannt ☐

**40. Schätzen Sie den Anteil der ärztlichen Mitarbeiter, die von Ihrer Abteilung zu**

**anderen Abteilungen umverteilt werden sollen (Angabe in %).**

**41. In welche Abteilungen wurden die Mitarbeiter umverteilt (Einzelnennungen, z. B.**

**Gastroenterologie , Intensivstation etc.)?**

Freitext

**42. Schätzen Sie den Anteil der ärztlichen Mitarbeiter Ihrer Abteilung in "Kurzarbeit"**

**oder "Schichtarbeit" (Angabe in %).**

**43. Schätzen sie den Anteil der ärztlichen Mitarbeiter Ihrer Abteilung die sich mit**

**SARS-CoV-2 infiziert haben (Angabe in %).**

**44. Wurde von Ihrer Klinikleitung Überstundenabbau angeordnet?**

Ja ☐

Nein ☐

Nicht bekannt ☐

**45. Wurde von Ihrer Klinikleitung Urlaub angeordnet?**

Ja ☐

Nein ☐

Nicht bekannt ☐

**46. Konnte ausreichend angemessene Schutzausrüstung für Ihr Personal zur**

**Verfügung gestellt werden?**

Ja ☐

Nein ☐

Nicht bekannt ☐

**47. Mussten Notfalloperationen aufgrund von krankheitsbedingtem Ausfall**

**verschoben bzw. verlegt werden?**

Ja ☐

Nein ☐

Nicht bekannt ☐

**48. Würden Sie zum aktuellen Zeitpunkt schätzen, dass ein Nichterreichen der**

**Zielvorgaben finanzielle Konsequenzen für Ihre Abteilung hat?**

Ja ☐

Nein ☐

Nicht bekannt ☐

**49. Würden Sie zum aktuellen Zeitpunkt schätzen, dass ein Nichterreichen der**

**Zielvorgaben personelle Konsequenzen für Ihre Abteilung hat?**

Ja ☐

Nein ☐

Nicht bekannt ☐

**50. Schätzen Sie den Verlust Ihrer Zielvorgaben für den Umsatz 2020 in %.** *(Sollte der Umsatz an Ihrer Klinik steigen oder gleich bleiben, so tragen Sie hier bitte eine "0" ein.)*

**51. Schätzen Sie den Verlust Ihrer Zielvorgaben für "Case Mix Punkte" in %.** *(Sollten die Case Mix Punkte an Ihrer Klinik steigen oder gleich bleiben, so tragen Sie hier bitte eine "0" ein.)*

**52. Schätzen Sie den Verlust Ihrer Zielvorgaben für "Case Mix Index" in %.** *(Sollte der Case Mix Index an Ihrer Klinik steigen oder gleich bleiben, so tragen Sie hier bitte eine "0" ein.)*

**Auswirkung der SARS-CoV-2-Pandemie auf die derzeitigen Fallzahlen**

**53. Die Anzahl an Notfalloperationen hat zugenommen (Angabe in %).** *(Sollte die Anzahl an Notoperationen abgenommen haben oder gleich geblieben sein, so tragen Sie hier bitte eine "0" ein.)*

**54. Die Anzahl an Notfalloperationen hat abgenommen (Angabe in %).** *(Sollte die Anzahl an Notoperationen zugenommen haben oder gleich geblieben sein, so tragen Sie hier bitte eine "0" ein.)*

**55. Die Anzahl an chirurgischen Notfällen in der Notaufnahme hat zugenommen**

**(Angabe in %).** *(Sollte die Anzahl an chirurgischen Notfällen in der Notaufnahme abgenommen haben oder gleich geblieben sein, so tragen Sie hier bitte eine "0" ein.)*

**56. Die Anzahl an chirurgischen Notfällen in der Notaufnahme hat abgenommen**

**(Angabe in %).** *(Sollte die Anzahl an chirurgischen Notfällen in der Notaufnahme zugenommen haben oder gleich geblieben sein, so tragen Sie hier bitte eine "0" ein.)*

**57. Die Anzahl an onkologischen Patienten in der Sprechstunde hat zugenommen**

**(Angabe in %).** *(Sollte die Anzahl an onkologischen Patienten in der Sprechstunde abgenommen haben oder gleich geblieben sein, so tragen Sie hier bitte eine "0" ein.)*

**58. Die Anzahl an onkologischen Patienten in der Sprechstunde hat abgenommen**

**(Angabe in %).** *(Sollte die Anzahl an onkologischen Patienten in der Sprechstunde zugenommen haben oder gleich geblieben sein, so tragen Sie hier bitte eine "0" ein.)*

**59. Haben Sie den Eindruck, dass Patienten aus Sorge vor einer SARS-CoV-2-**

**Infektion elektive Operationen absagen?**

Ja ☐

Nein ☐

Nicht bekannt ☐

**Ausblick für die Zeit nach der SARS-CoV-2-Pandemie**

**60. Die Zahl der Operationen wird nach der SARS-CoV2-Pandemie zunehmen.**

(1) Stimme voll zu (2) (3) (4) (5) Stimme überhaupt nicht zu

**61. Pflegekräfte werden zukünftig besser bezahlt werden.**

(1) Stimme voll zu (2) (3) (4) (5) Stimme überhaupt nicht zu

**62. Ärztliche Mitarbeiter und Mitarbeiterinnen werden zukünftig besser bezahlt**

**werden.**

(1) Stimme voll zu (2) (3) (4) (5) Stimme überhaupt nicht zu

**63. Ihre Abteilung wird aufgrund der SARS-CoV2-Pandemie in Zukunft an**

**Bedeutung verlieren.**

(1) Stimme voll zu (2) (3) (4) (5) Stimme überhaupt nicht zu

**64. Ihre Abteilung geht insgesamt geschwächt aus der SARS-CoV-2 Pandemie**

**hervor.**

(1) Stimme voll zu (2) (3) (4) (5) Stimme überhaupt nicht zu

**65. Ihre Abteilung geht strukturell geschwächt (d.h. mit weniger Betten) aus der**

**SARS-CoV-2 Pandemie hervor.**

(1) Stimme voll zu (2) (3) (4) (5) Stimme überhaupt nicht zu

**66. Ihre Abteilung geht personell geschwächt aus der SARS-CoV-2 Pandemie**

**hervor.**

(1) Stimme voll zu (2) (3) (4) (5) Stimme überhaupt nicht zu

**67. Hier können Sie Anmerkungen zur aktuellen Situation als Freitext hinterlegen. Teilen Sie uns gerne auch Anmerkungen und Feedback mit.**

Freitext
