# Supplementary figures and images for: Effects of the SARS-CoV-2 pandemic on surgery – a national cross-sectional study
Source: Chirurg. 2020 Aug 10;91(9):762–8. [Article in German] doi: 10.1007/s00104-020-01256-x (PMC7416587; doi:10.1007/s00104-020-01256-x)

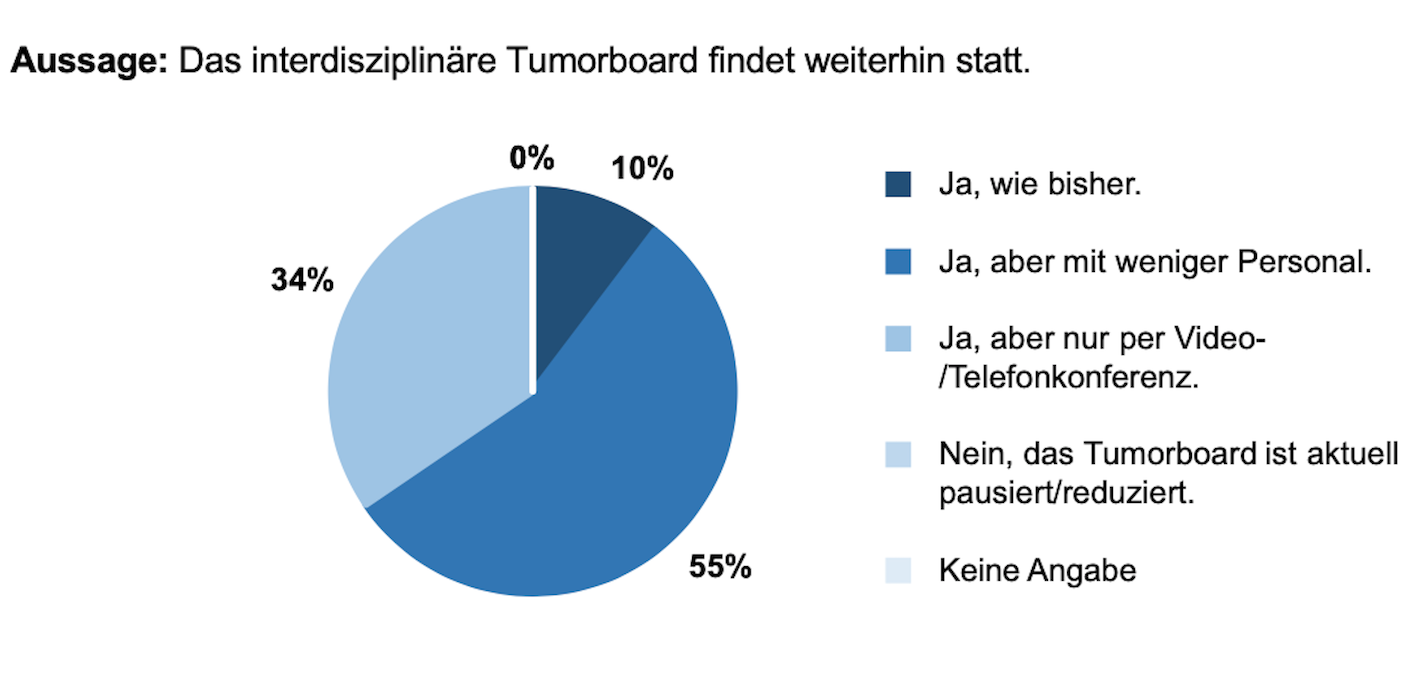

Supplement: Supplementary file 2 [file 104_2020_1256_MOESM2_ESM.tiff]
